# A Case of Omphalitis Revealing Alloimmune Neonatal Neutropenia

**DOI:** 10.7759/cureus.44409

**Published:** 2023-08-30

**Authors:** Nasa Machimoto, Yosuke Baba, Yuri Takaoka, Hiromichi Shoji, Toshiaki Shimizu

**Affiliations:** 1 Department of Pediatrics, Juntendo University Shizuoka Hospital, Shizuoka, JPN; 2 Department of Pediatrics, Juntendo University Faculty of Medicine, Tokyo, JPN

**Keywords:** alloimmune neonatal neutropenia, human neutrophil alloantigen, term neonate, clinical approaches & management, autoimmune neutropenia

## Abstract

Neutropenia, characterized by a decrease in peripheral blood neutrophil count less than 1500/µL, poses significant clinical challenges due to its association with recurrent infections. This paper presents a rare and intriguing case of alloimmune neonatal neutropenia (ANN), an uncommon variant of neutropenia instigated by the transplacental transfer of maternal anti-neutrophil antibodies that consequently induce opsonization and phagocytosis of the neonate’s neutrophils within the reticuloendothelial system.

The patient, an 18-day-old boy, was born at 36 weeks five days of gestation and weighed 2465 g, an attribute considered appropriate for gestational age (AGA). He experienced multiple episodes of skin and respiratory infections, coupled with delayed umbilical cord separation and demonstrated a significant reduction in neutrophil count. Despite these symptoms, the patient did not develop bacteremia and his condition improved with antibiotic therapy, leading to his discharge from the hospital. Crucially, both the patient and his mother tested positive for anti-HNA (human neutrophil alloantigen)-1a and anti-HNA-1b antibodies, indicative of a diagnosis of ANN.

ANN is intriguing in its clinical course, where despite neutropenia, severe infections are relatively uncommon, and the majority of cases resolve spontaneously within several months post-birth as the maternal antibodies diminish. Nevertheless, there have been reports of moderate to severe infections, demanding clinical intervention and close patient monitoring. The patient in our case was treated with prophylactic antibiotics for six weeks, until a rise in neutrophil count was confirmed, stemming from the severity and recurrence of infections.

The issue of using antibiotics and granulocyte colony-stimulating factor (G-CSF) agents in the treatment of ANN remains contentious, with contrasting reports regarding their efficacy and safety. The balance between the prospective therapeutic advantages, potential risks such as antibiotic resistance, and the possibility of inducing leukemia with long-term administration of G-CSF agents necessitates meticulous deliberation.

This case underscores the crucial role of early recognition of ANN in neonates presenting with neutropenia. Prompt diagnosis enables a more targeted approach to treatment, reduction in unnecessary antibiotic administration, and specific testing, thus impacting the overall patient management and potentially improving outcomes. Furthermore, in the event of delayed umbilical cord separation in neonates, healthcare providers should consider ANN and other immunodeficiencies related to neutrophil functional abnormalities as potential diagnoses. This patient's story accentuates the need for further investigations to elucidate the precise etiology and pathogenesis of ANN, paving the way for improved diagnostic tools and effective therapeutic strategies.

## Introduction

Alloimmune neonatal neutropenia (ANN) is an under-recognized and complex condition that arises during the intricate biological process of pregnancy. It emerges when the maternal immune system becomes sensitized to the human neutrophil alloantigen (HNA), which is derived from the paternal genome. This leads to the production of specific antibodies against mature fetal neutrophils. These antibodies can cross the placental barrier and induce a state of neutropenia in the newborn. Although these antibodies typically disappear within a few months, leading to a resolution of the neutropenia, the potential implications of ANN on neonatal health must not be overlooked. It becomes essential to consider the possibility of ANN when managing neonates with unexplained neutropenia.

Pregnancy-associated immunological changes and their impact on both mother and child's health is an area of ongoing research. Of particular interest is the production of anti-neutrophil antibodies. Studies suggest that the prevalence of these antibodies during pregnancy is approximately 19.6%, highlighting the relevance of this phenomenon [1]. Nevertheless, the true incidence of ANN, particularly in Japan, remains unclear. This is possibly due to a combination of factors such as spontaneous resolution of some cases, non-specific clinical presentation, and the challenges in diagnostic modalities, leading to missed diagnoses.

Over the years, a multitude of treatment strategies for ANN have been proposed, reflecting the diverse clinical presentations and complexities associated with this condition. Despite these efforts, no singularly effective treatment has been definitively established.

In this article, we recount our encounter with a perplexing case of a neonate who presented with omphalitis and unexplained neutropenia, eventually diagnosed as ANN. A positive test result for anti-neutrophil antibodies led us to this diagnosis, following which the patient's condition spontaneously resolved after approximately two months of observation.

Through the course of this report, we provide a comprehensive discussion on the clinical course of this patient, our diagnostic approach, the inherent complexities of managing ANN, and the potential strategies for improving neonatal outcomes. Our goal is to raise awareness about ANN among clinicians and to promote the formulation of better diagnostic and treatment strategies through ongoing research and clinical discussions.

## Case presentation

An 18-day-old boy presented with a main complaint of fever and redness in the umbilical region. His family history revealed no incidence of hematologic malignancies, immunodeficiency syndrome, or sudden death among his blood relatives. The maternal history included gravida 4, para 2, with a history of two artificial abortions with the current husband. There were no infections, no history of pharmaceutical use, and no gestational hypertension. The patient's medical history noted that he was delivered via natural vaginal delivery at 36 weeks and five days of gestation, weighed 2456 g at birth, and had Apgar scores of eight at one minute and nine at five minutes. History of current condition: the infant was active after birth and was nursing well, but the mother noticed redness around the umbilical region when the infant was 14 days old and went to her local physician. The condition was diagnosed as umbilical granuloma. The umbilicus was treated, and the infant’s condition was monitored. However, he became irritable and developed a fever with a body temperature of 38°C since 17 days of age. The fever persisted the following day, and the irritability worsened. Thus, he was brought to our department as an emergency outpatient for consultation. Accordingly, he was admitted for detailed examination and treatment.

Vital signs and physical examination findings are as follows: condition at admission: weight, 2,892 g (-0.3SD); body temperature, 37.8°C; heart rate, 140 bpm; respiratory rate, 40 breaths/min; blood pressure, 83/42 mmHg. Head: flat anterior fontanelle and good facial color. Pharynx: no redness. Neck: no swollen lymph nodes. Chest: breath and heart sounds are clear. Abdomen: soft, no enhanced or weakened bowel sounds, and no liver enlargement. Redness and heat in the umbilical region and surrounding area; the umbilical cord is still attached. Limbs: no edema, capillary refill time: within one second. Skin: no rash. Other: no apparent external malformation. Chest and abdominal radiographs at admission: no infiltrative shadows in the hilar area and no enlargement of the heart, but with a thymus shadow. Laboratory findings at admission (Table 1): white blood cell count was 10,700/µL, but the neutrophil count was <500L/µL, indicating neutropenia, and the monocyte count was elevated. No reduction in any other hematopoietic cells was observed. No apparent abnormalities such as hemolysis were found on biochemical tests. Immunological tests showed that the c-reactive protein (CRP) level was elevated at 4.6 mg/dL, but the IgG, IgA, and IgM levels were all within the normal range.

**Table 1:** Blood test findings on admission Neutrophil levels were found to be decreased to 107/µL. The elevation of CRP suggested an infection. However, no other abnormalities were detected in biochemical or immunological findings. WBC, white blood cell count; RBC, red blood cell count; HB, hemoglobin; HT, hematocrit; PLT, platelet count; CRP, c-reactive protein; Ig, immunoglobulin; TP, total protein; Alb, albumin; AST, aspartate aminotransferase; ALT, alanine aminotransferase; LDH, lactate dehydrogenase; γ-GTP, gamma-glutamyl transferase; T-Bil, total bilirubin; CPK, creatine phosphokinase; UA, uric acid; BUN, blood urea nitrogen; CRE, creatinine; BS, blood sugar

Test	Result		Reference Values
Blood cell counts			
WBC	10,700	/μL	9,000-30,000
Neutrophils	1	%	50-70
Lymphocytes	64	%	30-50
Monocytes	27	%	5-10
Eosinophils	6	%	1-5
Basophils	0	%	0-1
Atypical Lymphocytes	0	%	0
Blasts	0	%	0
Promyelocytes	0	%	0
Myelocytes	2	%	0
Metamyelocytes	0	%	0
RBC	429×10^4^	/μL	430-567
HB	14.9	g/dL	13.4-17.1
HT	44.8	%	40.4-51.1
PLT	35.5×10^4^	/μL	15.3-34.6
Serum			
TP	6.2	mg/dL	6.5-8.5
Alb	3.4	mg/dL	4.0-5.2
AST	23	IU/L	5-37
ALT	12	IU/L	6-43
LDH	233	IU/L	119-221
γ-GTP	241	IU/L	0-75
T-Bil	2.2	mg/dL	0.4-1.2
CPK	51	IU/L	57-240
Na	136	mEq/L	135-145
Cl	98	mEq/L	96-107
K	5.3	mEq/L	3.5-5.0
UA	1.6	mg/dL	3.5-6.9
BUN	5.8	mg/dL	9-21
Cre	0.19	mg/dL	0.6-1.0
BS	77	mg/dL	50-90
Immunological			
CRP	4.6	mg/dL	<0.02
IgG	752	mg/dL	200-600
IgA	22	mg/dL	1-4
IgM	39	mg/dL	15-70

**Figure 1:**
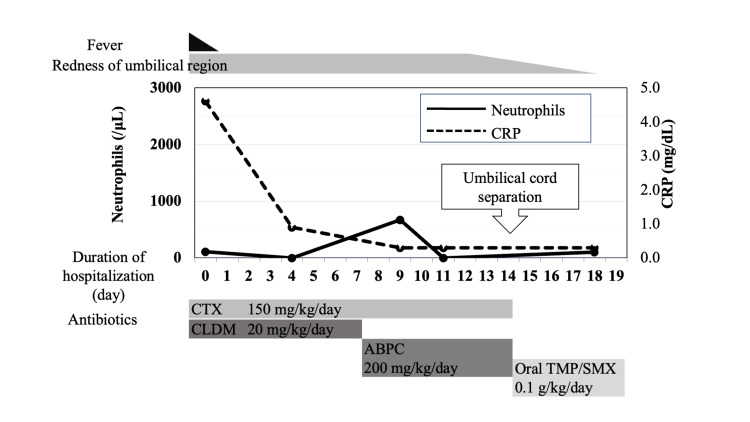
Clinical course after admission Provisional diagnoses of omphalitis and congenital neutropenia were made based on the physical examination and blood test findings after admission. Blood cultures were negative, but intravenous antimicrobials were administered for two weeks, followed by oral TMP/SMX as a prophylactic treatment. CRP, c-reactive protein; CTX, cefotaxime sodium; ABPC, ampicillin; CLDM, clindamycin; TMP/SMX, sulfamethoxazole-trimethoprim

We performed a blood culture, urine quantitative culture, and skin culture peripheral to the umbilicus. The course of treatment following admission is depicted in Figure 1. We started antibiotic treatment, considering bacterial infection as a possible cause of the fever and elevated inflammatory response. He was active, without abnormal neurological findings, such as bulging fontanelle or lethargy, indicating no clear signs of meningitis. Additionally, the source of infection was thought to be localized to the umbilical cord. Therefore, considering the normal neurological examination and the identified local cause, a lumbar puncture (LP) to rule out meningitis was not performed for this case. As the initial antibiotic treatment of omphalitis in neonates, cefotaxime (150 mg/kg/day) and clindamycin (20 mg/kg/day) were administered based on the possibility of the infection being caused by *Escherichia coli*, *Staphylococcus aureus*, or anaerobic bacteria. The fever remitted immediately after treatment was started, and blood tests revealed that the inflammatory response was reduced, but no increase in neutrophil count was observed. Abdominal ultrasonography and simple abdominal magnetic resonance imaging were performed during hospitalization to ascertain the cause of the delayed umbilical cord separation and the omphalitis, but the scans showed no urachal remnant or any intra-abdominal or subcutaneous tissue abnormalities. The blood culture was negative for bacteriological cultures, but *Enterococcus faecalis* was detected in the skin and urine quantitative cultures. The condition was not diagnosed as a urinary tract infection, but we changed the antibiotic to ampicillin (200 mg/kg/day) based on the causative bacteria of omphalitis. The redness in the umbilical region persisted, but CRP became negative on day nine of hospitalization, and the umbilical cord separated on day 14 of antibiotics. Thereafter, the redness in the umbilical region was eased. Antibiotics were administered for 15 days, which completed the treatment of the omphalitis. However, the blood test results continued to indicate a reduced neutrophil count; therefore, we conducted peripheral blood lymphocyte fractionation by using flow cytometry and a neutrophil function test to investigate neutropenia. No abnormalities were found with the lymphocyte fractionation. However, in the neutrophil function test, we found that while the gating strategy could not be used for almost all of the neutrophils, no apparent abnormalities in terms of phagocytic and bactericidal capacities were found based on the reference value. In the histogram analysis of peripheral blood leukocytes, the findings led us to suspect granulocyte destruction due to a large number of unevenly sized cells with a high degree of granule density. These results indicated immunological neutropenia caused by anti-neutrophil antibodies. Hence, we suspected ANN and tested for anti-neutrophil antibodies in both the mother and this patient. His status was good, but considering the risk of bacterial infection associated with persistent neutropenia, we decided to administer sulfamethoxazole-trimethoprim (TMP/SMX) drug (0.1 g/kg/day). He was discharged on hospital day 18.

Our policy is to administer granulocyte colony-stimulating factor (G-CSF) and examine the bone marrow when neutropenia is prolonged, typically considered in cases of severe or symptomatic neutropenia persisting beyond a certain timeframe, such as one to two weeks, or in those at significant risk of infection, in line with our clinical practice guidelines. In the antibody test, both the mother and he tested positive for anti-HNA1-a antibodies and anti-HNA1-b antibodies, which led to a definitive diagnosis of ANN. The patient visited the hospital as an outpatient, continued receiving TMP/SMX drug and underwent regular monitoring for the progress of the neutrophil count. He visited the hospital as an outpatient, continued receiving TMP/SMX drugs, and underwent regular monitoring for the progress of the neutrophil count. From 50 days of age, his neutrophil count began to increase, without any interim infection; therefore, the oral TMP/SMX drug treatment was discontinued at 60 days of age. He is currently being monitored on an outpatient basis, with check-ups approximately once every three months, and the monitoring has been ongoing for the first ten months of his life. He has had no recurrence of neutropenia.

## Discussion

Neutropenia is defined when the peripheral blood neutrophil count is less than 1,500/µL, with 500-1,000/µL classified as moderate and less than 500/µL classified as severe. While some authors claimed that the risk of infection increases when the count is less than 1,000/µL, others suggest that this level is not problematic provided that the bone marrow production capability is normal. The causes of a reduced neutrophil count include neutrophil production disorders, ineffective erythropoiesis or dysfunctional neutrophil mobilization from the bone marrow to the blood, neutrophil distribution disorder in the body, or accelerated consumption or destruction of neutrophils in the peripheral blood [2-5]. The clinical symptoms of a reduced neutrophil count often present as recurrent respiratory infections, skin infections, otitis media, stomatitis, and/or gingivitis.

The diagnosis in this case was ANN. The cause of ANN is the movement of anti-neutrophil antibodies across the placenta to him. This patient’s neutrophils then undergo opsonization and are then phagocytosed in the reticuloendothelial system, resulting in a reduced neutrophil count. HNA, a neutrophil-specific antigen for the same disease, is present as autoantigens, ranging from HNA-1 to HNA-5. HNA-1 is expressed on FcγRIIIb, is commonly involved in immunological neutropenia, and has the allotypes 1a, 1b, and 1c antigens. The main combinations are 1a/1a, 1a/1b, and 1b/1b; but occasionally, HNA-1 null is present, which lacks both the 1a and 1b antigens. The incidence of HNA-1 null is said to be 0.15%, with few reported cases [3]. ANN occurs during pregnancy when the mother does not have human neutrophil antigens for anti-HNA derived from the father, so the mother then produces HNA antibodies. These antibodies then move across the placenta to the fetus, reducing the neutrophil count. Repeated abortions are said to increase the possibility of the mother producing anti-HNA antibodies [4]. In this case, both the mother and the affected child were positive for anti-HNA-1a and anti-HNA-1b antibodies. However, the mother did not have a reduced neutrophil count, so we think the mother’s HNA was HNA-1 null. Neutrophil antibodies are detected in approximately 20% of pregnancies, but ANN is reported to occur in 0.2% of births [5]. However, given that reports indicate that reduced neutrophil counts are observed in 8% of cases admitted to the neonatal intensive care unit, ANN is often diagnosed incidentally [6]. Additionally, some cases are not diagnosed when the condition is not associated with complications such as infection; hence, the incidence of the condition in Japan is still unknown.

Autoimmune neutropenia (AIN) immunologically causes a reduced neutrophil count in the same way as ANN but differs from ANN in that the reduced neutrophil count is caused by the self-production of anti-HNA antibodies. The major point of differential diagnosis is the onset timing. In ANN, the maternal antibodies present in the infant develop immediately after birth. By contrast, AIN commonly develops between three months to three years after birth. The presence or absence of the mother’s neutrophil antibodies may also be a point of differentiation. ANN is neutropenia that develops from the neonatal stage, and neutrophil counts may be moderately or severely reduced. However, it rarely causes serious infection, and many of the complicated cases are caused by skin infections (particularly omphalitis) and respiratory infections [7]. In addition, the neutrophil count increases at around three months after birth as the maternal antibodies disappear, and cases commonly resolve without treatment. However, given the reports of cases with moderate to severe infections such as bacteremia, the condition should be treated as needed and the patient’s progress should be carefully monitored [8].

In this case, separation of the umbilical cord was delayed, which is thought to have caused the omphalitis. The blood quantitative culture was negative but *E. faecalis* was detected from the skin culture in the umbilical region. Thus, this was thought to have been the etiological agent of the infection. Generally, *Staphylococcus aureus* is a common etiologic agent when neutrophils are reduced but gram-negative bacilli such as *E. coli* and Group B *streptococci*, which are frequently the etiological agents of infections in neonates, should also be considered. Prophylactic antibiotics have been reported to reduce the mortality rate of severe congenital neutropenia and neutropenia treated with cytotoxic therapy [9]. However, no clear policy has been implemented for the use of prophylactic antibiotics for ANN. Nevertheless, various differential diagnoses for neonatal neutropenia have to be considered (Table 2). In the present case, we started treatment with prophylactic administration of antibiotics considering the severe recurrent infection. The patient did not develop a severe infection during hospitalization, and the initial infection was relatively mild, which also led us to suspect ANN. Whether the disease does not generally require prophylactic administration of antibiotics is still controversial. Careful judgment is vital from the perspective of inducing resistant bacteria, among others.

**Table 2:** Differential diagnosis of neutropenia in newborns The causes of neutropenia are grouped into two main categories: causes extrinsic to marrow myeloid cells and intrinsic disorders of myeloid precursor cells. Each category includes various potential causes or conditions related to neutropenia. G6Pase, glucose 6-phosphatase; Ig, immunoglobulin; WHIM syndrome: warts, hypogammaglobulinemia, infections, and myelokathexis syndrome

Cause of neutropenia extrinsic to marrow myeloid cells	Intrinsic disorders of myeloid precursor cells
Infection	Cyclic neutropenia
Drug-induced	Severe congenital neutropenia
Immune neutropenia	Kostmann syndrome
Reticuloendothelial sequestration	Shwachman-Diamond syndrome
Bone marrow replacement	Dyskeratosis congenital
Cancer chemotherapy or radiation therapy	Chédiak-Higashi syndrome
Acquired disorder of myeloid cells	Griscelli syndrome, type Ⅱ
Aplastic anemia	p14 deficiency
Vitamin B12 or folate deficiency	Glycogen storage disease, type 1b
Acute leukemia	G6Pase, catalytic subunit 3, deficiency
Myelogenous leukemia	Barth syndrome
Myelodysplasia	Pearson’s syndrome
Prematurity with birth weight <2 kg	Common variable immunodeficiency
Chronic idiopathic neutropenia	IgA deficiency
Paroxysmal nocturnal hemoglobinuria	Severe combined immunodeficiency
	Hyper-IgM syndrome
	WHIM syndrome
	Cartilage-hair hyperplasia
	Schimke immune-osseous dysplasia

Opinions differ about the administration of G-CSF agents for neutropenia. Gilmore et al. reported that administration of G-CSF 5 µg/kg/day promotes neutrophil production and increases neutrophils by delaying apoptosis of granulocytes by antibodies, with the antigens themselves also being reduced [8]. In addition, the safety of administering G-CSF to neonates has been confirmed. Reports indicated that administration reduces both the rates of infection and mortality and shortens the length of hospital stays [10]. However, considering that this case may have spontaneously resolved and the risk of developing leukemia with long-term administration of G-CSF agents, careful administration is essential. G-CSF agents are indicated when ANN is complicated by severe infections or in cases of repeat infections. However, the patient’s status should be confirmed first based on, for example, bone marrow findings.

The detailed cause of delayed umbilical cord separation observed in this case and other ANN case reports has not been clarified. When the umbilical cord is pathologically detaching, neutrophils infiltrate from the peripheral region to the central part of the umbilicus. Thus, inflammation caused by neutrophilic infiltration is possible with umbilical cord separation. Delayed umbilical cord separation is also said to be caused by leukocyte adhesion deficiency, excessive leukocyte adhesion, and interleukin-1 receptor-associated kinase 4 deficiency [11]. Not only is cytotoxicity caused by neutrophilic infiltration but also chemokines such as CXCL8 and lipid mediators may also be involved. When separation of the umbilical cord is delayed in neonates, further detailed investigation should be performed with suspicion of these immunodeficiency disorders.

## Conclusions

In this report, we have detailed a case where ANN was identified in a neonate presenting with omphalitis. This case serves as an important reminder of the diverse range of pathologies that can present as neutropenia in neonates. While conditions such as infections or drug-induced neutropenia may be more common, the possibility of less frequent causes like ANN should not be overlooked.

Particularly in instances of delayed umbilical cord separation, healthcare professionals should consider the potential for underlying immunodeficiencies, which could be a result of abnormalities in neutrophil function. Our case suggests that such diagnoses can easily be missed due to the rarity of these conditions and the common occurrence of neutropenia in the neonatal period due to other causes.

By identifying and diagnosing these conditions early, we can significantly reduce the administration of unnecessary antibiotics, thus mitigating the risk of antibiotic resistance development. Moreover, early diagnosis allows for the more timely initiation of appropriate interventions and potentially avoids the need for additional treatments and tests that may place unnecessary burdens on the healthcare system and the patient. This case highlights the need for maintaining a broad differential when assessing neutropenia in neonates and the potential implications of early and accurate diagnosis on patient management and healthcare resources.
